# Supplementary material for: Changes in the Bacterioplankton Community Structure from Southern Gulf of Mexico During a Simulated Crude Oil Spill at Mesocosm Scale
Source: Microorganisms. 2019 Oct 11;7(10):441. doi: 10.3390/microorganisms7100441 (PMC6843455; doi:10.3390/microorganisms7100441)
Supplement: Supplementary file 1 [file microorganisms-07-00441-s001.pdf]

Table S1. BLAST analysis of unassigned ASVs with differential abundances through the mesocosms experiment. R = reference sample.

ASV Internal ID	Sampling Day	Similarity (%)	E-value	Closest Hits (NCBI Accession Number)
**75422**	R, 0	92.5	$2.37e^{-91}$	*Lewinella cohaerens* (NR_115012.1)
**f8a67**	R, 0, 2, 4, 6	98.37	$4.93e^{-118}$	*Prochlorococcus marinus* (NR_125480.1)
**43959**	R, 0, 2, 4, 6	96.80	$1.38e^{-113}$	*Angustibacter luteus* (NR_112956.1)
**03b6d**	R, 0	97.20	$2.96e^{-115}$	*Ilumatobacter fluminis* (NR_041633.1)
**23f4cf**	8, 10, 14	91.20	$3.07e^{-90}$	*Zymomonas mobilis* (NR_113634.1)
**6ce57**	R, 0	96.78	$4.96e^{-113}$	*Candidatus Pelagibacter ubique* (NR_074224.1)
		98.80	$6.33e^{-122}$	*Aquicoccus porphyridii* (NR_158155.1)
**15a9d**	2, 4, 6	98.80	$6.33e^{-122}$	*Maliponia aquimaris* (NR_149300.1)
		98.80	$6.33e^{-122}$	*Antarctobacter heliothermus* (NR_026406.1)
**420e0**	8, 10, 14	93.54	$3.92e^{-99}$	*Nisaea nitritireducens* (NR_043924.1)

Table 2. Concentrations of polycyclic aromatic hydrocarbons expressed in $\mu g \cdot L^{-1}$.

Compound	Reference	0	2	4	6	8	10	14
Napthalene	0.5883	0.9012	0.1314		0.0004			0.0004
2-Methylnaphthalene	0.5911	0.8514	0.1972	0.0014	0.0010			
1-Metilnaphtalene	0.5635	0.8954	0.2717	0.0015	0.0009			
Biphenyl	0.0136	0.0208	0.0261					
2,6-Dimethylnaphthalene	0.0621	0.0914	0.1936	0.0138	0.0021	0.0025	0.0023	0.0020
Acenaphthylene	0.0054	0.0063	0.0082	0.0037				
Acenaphtene	0.0031	0.0052	0.0122	0.0035				
2,3,5-Trimethylnaphthalene	0.0185	0.0266	0.1152	0.2082	0.0276	0.0244	0.0173	0.0062
Fluorene	0.0169	0.0276	0.1039	0.0321	0.0043	0.0069	0.0037	0.0033
Dibenzothiophene		0.0125	0.1932	0.3182	0.0436	0.0531	0.0523	0.0310
Phenantrene	0.0075	0.0147	0.1017	0.1679	0.0223	0.0252	0.0397	0.0248
Anthracene	0.0060	0.0063	0.0062	0.0166	0.0062	0.0062	0.0074	0.0069
1-Methylphenanthrene		0.0069	0.0276	0.2711	0.0395	0.0476	0.1017	0.1314
Fluoranthene		0.0028	0.0028	0.0062	0.0034	0.0036	0.0043	0.0052
Pyrene			0.0012	0.0395	0.0078	0.0101	0.0206	0.0262
Chrysene		0.0037	0.0039	0.0607	0.0125	0.0168	0.0363	0.0602
Benzo[*b*]fluoranthene		0.0024	0.0023	0.0098	0.0031	0.0036	0.0069	0.0096
Benzo[*e*]pyrene				0.0251	0.0060	0.0076	0.0154	0.0230
Benzo[*a*]pyrene			0.0033	0.0057	0.0035	0.0035	0.0042	0.0041
Dibenz[*a,h*]anthracene				0.0054			0.0047	0.0047
Benzo[*ghi*]perylene				0.0111	0.0044	0.0043	0.0072	0.0093
